# Treatment of Opioid Use Disorder in Canadian Psychosocial Addiction Programs: A National Survey of Policy, Attitudes, and Practice

**DOI:** 10.1177/07067437221082858

**Published:** 2022-03-08

**Authors:** David C. Hodgins, Mathew Budd, Gail Czukar, Simon Dubreucq, Lois A. Jackson, Brian Rush, Lena C. Quilty, Denise Adams, T. Cameron Wild

**Affiliations:** 1 Department of Psychology, 192287 University of Calgary, Calgary, Alberta; 2 Addictions and Mental Health Ontario, Toronto, Ontario; 3 Department of Psychiatry, 25443 Centre Hospitalier de l’Université de Montréal, Montréal, Québec; 4 School of Health and Human Performance, Faculty of Health, Dalhousie University, Halifax, Nova Scotia; 5 Centre for Addiction and Mental Health, Toronto, Ontario; 6 Campbell Family Mental Health Research Institute, Centre for Addiction and Mental Health; Department of Psychiatry, University of Toronto, Toronto, Ontario; 7 School of Public Health, 3158 University of Alberta, Edmonton, Alberta

**Keywords:** opioid use disorder treatment, opioid agonist treatment, naloxone, addiction services

## Abstract

**Objective:**

To describe current approaches in treatment of opioid use disorder (OUD) within Canadian psychosocial outpatient, day, and residential addiction treatment programs, with an emphasis on the use of opioid agonist therapy (OAT).

**Method:**

An online census survey was conducted in English and French of Canadian psychosocial addiction treatment programs (*N* = 214).

**Results:**

Programs estimated that 25% of their clients have OUD. A slight majority of programs provide some type of specialized services to clients with OUD (58%), most frequently providing or facilitating access to OAT but also specialized counselling, case management, education, and harm reduction services.

Most programs reported that they admitted clients on OAT (88%) and only a minority expected or encouraged clients to taper (14%) or discontinue (6%). Programs focusing on client abstinence as the treatment goal were more likely to expect or encourage tapering or discontinuation than programs that focus on helping clients achieve personal consumption goals. Of programs that did not currently facilitate OAT, 44% indicated that they would provide OAT, but lacked the necessary accreditation, physician support, or other resources. No philosophical objections to OAT were noted.

OAT initiation was provided by 30% of programs, 23% referred to another service within their organization, and 29% referred to a service outside their organization. The remaining 18% did not facilitate OAT initiation at all, ranging from 0% in Quebec to 23% in the Prairies. Overdose response kits were provided by 86% of programs. The majority not providing kits indicated willingness if policy support and resources were provided (67%).

**Conclusions:**

Overall, the results demonstrate that psychosocial programs provide some specialized services for OUD but desire further support specifically to provide OAT, including training, knowledge, and the expertise of individuals qualified to prescribe and dispense OAT. Many psychosocial treatment programs expressed a need for staff and resources for this purpose.

## Abrégé

**Objectif:**

Décrire les approches actuelles du traitement du trouble lié à l’utilisation d’opioïdes (TUO) dans le cadre des programmes canadiens de traitement de la dépendance psychosociale qu’ils soient de jour ou résidentiels, avec un accent mis sur le recours à la thérapie des agonistes opioïdes (TAO).

**Méthode:**

Une enquête de recensement en ligne a été menée en anglais et en français des programmes canadiens de traitement de la dépendance psychosociale (N = 214).

**Résultats:**

Les programmes estimaient que 25% de leurs clients ont un TUO. Une légère majorité des programmes offre un certain type de services spécialisés aux clients souffrant du TUO (58%), le plus souvent en offrant ou en facilitant l’accès à la TAO, mais aussi à une consultation spécialisée, à la gestion de cas, à l’éducation, et aux services de réduction des méfaits.

La plupart des programmes rapportaient admettre des clients à la TAO (88%) et seule une minorité encourageait les clients à diminuer (14%) ou discontinuer (6%), ou s’y attendait. Les programmes axés sur l’abstinence des clients comme but du traitement étaient plus susceptibles de prévoir ou d’encourager la diminution ou la discontinuité que les programmes qui mettent l’accent sur aider les clients à atteindre leurs buts de consommation personnelle. Sur les programmes qui ne dispensaient pas présentement la TAO, 44% indiquaient qu’ils offriraient la TAO, mais qu’il leur manquait l’accréditation nécessaire, le soutien d’un médecin ou d’autres ressources. Aucunes objections philosophiques à la TAO n’ont été notées.

L’initiation de la TAO a été fournie par 30% des programmes, 23% ont référé à un autre service au sein de leur organisation, et 29% ont référé à un autre service en dehors de leur organisation. Les 18% restants n’ont pas du tout dispensé l’initiation de la TAO, de 0% au Québec à 23% dans les Prairies. Des trousses d’intervention pour surdose ont été fournies par 86% des programmes. La majorité qui ne les a pas fournies a indiqué y être disposée si on leur fournissait un soutien politique et des ressources (67%).

**Conclusions:**

En général, les résultats démontrent que les programmes psychosociaux offrent des services spécialisés pour le TUO mais qu’ils désirent plus de soutien spécifiquement pour offrir la TAO, notamment une formation, des connaissances, et l’expertise de personnes compétentes pour prescrire et dispenser la TAO. Nombre de programmes de traitements psychosociaux ont exprimé le besoin de personnel et de ressources à cette fin.

## Introduction

The opioid crisis^1,2^ has challenged the Canadian health system to enact rapid, actionable policy responses to scale up evidence-based prevention, harm reduction, and treatment services. In Canada and elsewhere, two types of programs have provided treatment for people with opioid use disorder (OUD): opioid agonist treatment (OAT) programs that provide maintenance pharmacotherapies (typically methadone and buprenorphine), usually delivered via specialty outpatient medical services; and, addiction treatment and recovery programs that provide a variety of non-pharmacologic psychosocial interventions offered via non-residential outpatient or drop-in, day/evening and residential services. In some communities, those who use opioids can access either or both care options, whereas many other communities likely have limited access to OAT. Historically, OAT and psychosocial treatment programs have operated largely independently, but the opioid crisis has increased recognition of gaps between these services and the potential for better integration between them. This report describes treatment program attitudes, policies, and practices for treating clients presenting with OUD among Canadian specialty addiction services offering psychosocial programming. It does not attempt to report on the effectiveness of these psychosocial treatments.

Evidence consistently supports the use of OAT for improving patient retention, reducing morbidity and mortality, and/or reducing the risk of comorbid infectious diseases in various contexts.^3^ In Canada, buprenorphine-naloxone is recommended for first-line treatment due to its high therapeutic index, low risk of overdose and interactions with other medications, and less restrictive prescription policies.^4–6^ Methadone is recommended for those who respond poorly to buprenorphine and naloxone, and may be indicated depending on various patient factors, including comorbidity, treatment history, severity of withdrawal and/or dependence symptoms, and patient preference.^3^

Current practice guidelines indicate that OAT should be routinely offered alongside standard clinician-level medical support and unstructured counselling. Evidence is mixed regarding whether linkage to structured psychosocial treatment for OUD in conjunction with OAT improves outcomes.^7,8^ A recent review concluded that addition of optimal combinations of psychosocial treatment options to OAT may be beneficial for certain client populations,^8^ notably, clients with comorbid addictions and mental health conditions.^9–11^

Despite almost 50 years of research, relatively little is known about the effectiveness of non-pharmacological psychosocial treatment for OUD.^12^ Yet, many patients with OUD enter addiction treatment programs offering only psychosocial interventions. Historically, these programs subscribed to a strict abstinence policy, which precluded and discouraged concurrent and post-treatment use of OAT. However, the ongoing opioid emergencies in Canada and elsewhere have encouraged a re-evaluation of these attitudes and practices but the extent of change is not clear. Therefore, the broad goal of this research was to describe current approaches to the treatment of OUD within Canadian psychosocial addiction treatment programs. Our specific research objectives were to: (1) describe the proportion of programs offering specialized treatment for OUD clients, including the provision of OAT directly or through referral; (2) describe the programs’ policies and procedures regarding OAT provision; (3) compare abstinence-only focused programs to programs supporting flexible client consumption goals on access to OUD-relevant services; and (4) describe the proportion of programs that provided access to overdose response kits (i.e., naloxone).

## Methods

### Study Design

The study was an observational, cross-sectional, Canada-wide census survey of publicly-funded and privately-operated Canadian psychosocial addiction treatment programs. Specifically, we recruited key informants (i.e., program mangers) to complete structured items describing their programs via an online survey offered in both English and French. The study was approved by the University of Calgary Conjoint Faculties Research Ethics Board (Certification #REB18-2067), and, as required, certificates of approval were obtained in British Columbia (BC Mental Health & Substance Use Services), Alberta (Alberta Health Services Provincial Research Administration, the Northern Alberta Clinical Trials and Research Centre for the Edmonton Zone, and Covenant Health Research Centre for the Covenant Health network), and Quebec (Centres Intégrés Universitaires De Santé Et De Services Sociaux and per-site where required). Additional administrative approvals were required for Nova Scotia Health and for privately-operated programs.

### Enumerating Canadian Psychosocial Treatment Programs

Comprehensive lists of programs operating in the 13 Canadian provinces and territories were compiled in collaboration with addiction policy leaders in each jurisdiction. National networks of private addiction treatment providers were also enumerated through personal communications with their representatives. These lists were supplemented through online scans of program databases and snowball nomination No programs operating in the Northwest Territories and Nunavut that satisfied the program eligibility criteria were located. This process resulted in a national list of 740 programs that were assessed for eligibility for the current study.

### Program Eligibility

Eligible programs were defined as specialty services offering non-pharmacological treatments for people with substance use disorders. Treatment was broadly defined and included recovery-oriented programs and those focusing solely on reduction or elimination of substance use.^13^

Independently practicing practitioners were excluded, as enumerating these services on a national scale was not feasible. In addition, the following services were ineligible: (1) programs employing fewer than two full-time equivalent care providers; (2) programs offering only primary care; transitional housing services not including a therapeutic or recovery component; (3) programs operating as Indigenous health services; (4) programs providing *only* detoxification or withdrawal management services and programs primarily providing harm reduction services, such as safe consumption sites; (5) programs primarily serving children and adolescents. The programs in 3, 4, and 5 above are the focus of other linked projects of CRISM's Implementation Science program (https://crism.ca/projects/implementation).

### Recruitment and Survey Administration

**Figure 1.**
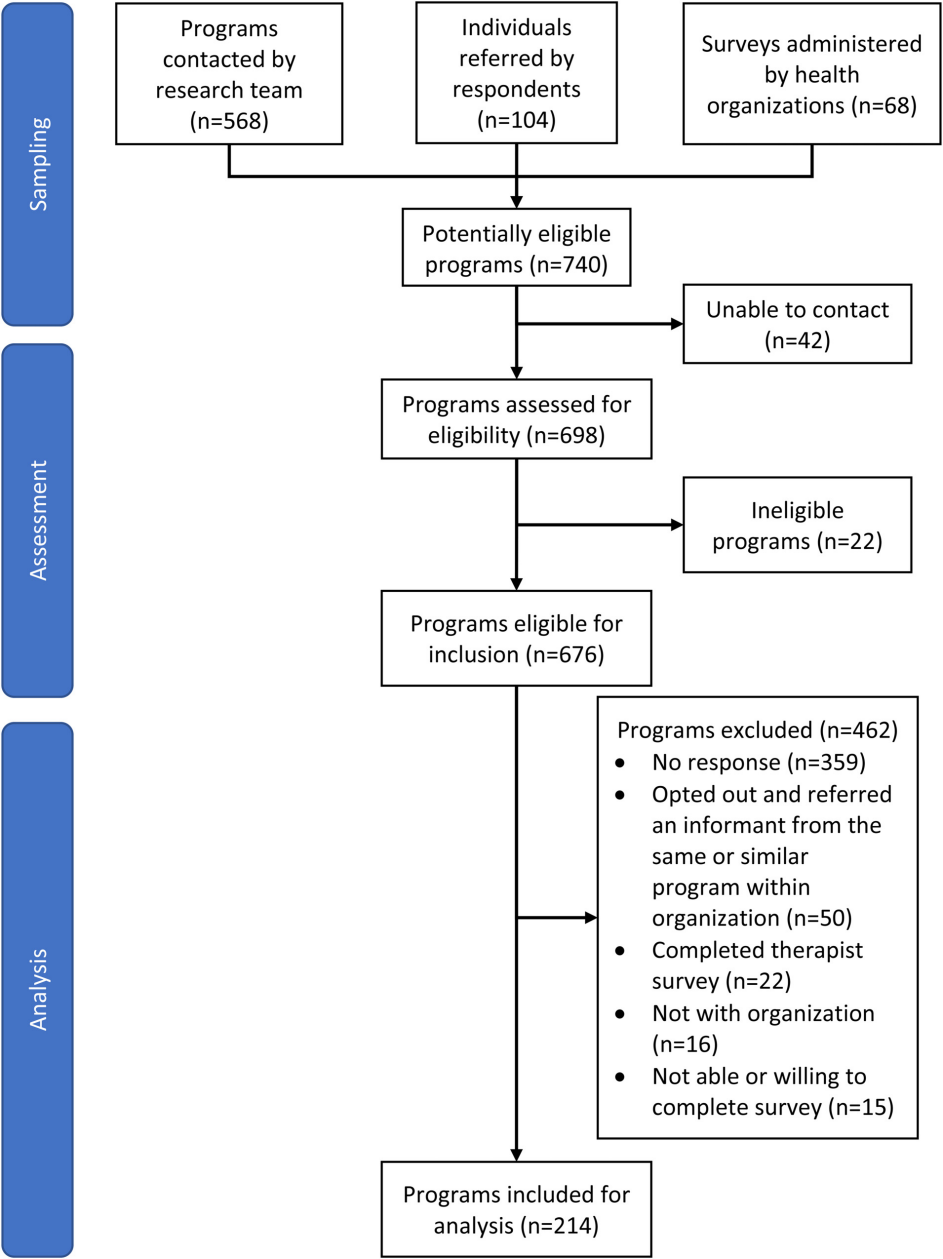
Program recruitment.

Of the 740 enumerated programs, 42 could not be contacted (Figure 1). Eligibility for inclusion of the remaining 698 programs was assessed through stakeholder consultation and personal communications with organizational management, and 22 were deemed ineligible. Programs were contacted via email to identify potential key informants. Those interested were provided with a project description, consent form, and survey link. Exceptions to this process occurred in Alberta and Quebec, where policy required that provincial health administrators contact potential programs on our behalf. Data were collected between March 2019 and March 2020.

As shown in Figure 1, 97% (676/698) of programs met all inclusion and did not meet exclusion criteria and 214 surveys were completed. The overall response rate was 27% (169/618; calculation excludes Quebec and Alberta, where survey administration procedures precluded quantifying the number of programs contacted).

### Survey Content

The key informant (program mangers) online survey was designed to collect descriptive data on individual programs and was drafted and pilot-tested in consultation with eligible Alberta-based health service providers and the project advisory group (Supplemental material). Survey items were modified from earlier program surveys^13,14^. The survey contained 87 items in 10 sections: (a) program and respondent identifiers [9 items]; (b) treatment details such as substance and behavioral addictions treated, specialized treatments for clients with OUD, and beliefs and practices [9 items]; (c) affiliation with OAT programs, policy, and practices with regards to OAT administration, barriers to and facilitators to providing OAT on-site [13 items]; (d) policies and practices related to naloxone kits [8 items]; (e) program operations such as client groups served and bio-sample testing [9 items]; (f) types of services and therapies [2 items]; (g) estimates of clientele served [14 items]; (h) philosophy and goals of services [8 items]; (i) admission and discharge policies [8 items]; and (j) outcome monitoring [7 items].

### Data Analyses

Descriptive analyses were conducted. For exploration of regional differences, the three prairie provinces were combined, as were the four Atlantic provinces. For proportions, the denominator excluded missing and “unsure” responses. Where relevant, the numbers of missing and unsure responses and the corrected denominator are reported. Binomial confidence intervals (90%) were calculated.^15^ Missing data rates per item were minimal (0% to 7%). Due to the length of the survey, some participants discontinued toward the end. A total of n = 184/214 key informants (86%) responded to the final item on the survey, although completion rates varied by question.

## Results

### Program Characteristics

Programs offered one or more of three levels of treatment: non-residential outpatient and walk-in services were offered by 128 programs (60%, n = 10 unsure, n = 21 missing); day and evening services (e.g., intensive non-residential service offered 3-4 h per day) were offered by 101 programs (47%, n = 4 unsure, n = 23 missing); and residential services were offered by 100 programs (48%; n = 2 unsure, n = 24 missing). Most programs served primarily an urban population (86%) and were funded either primarily or in part by provincial/territorial health authorities (n = 186/197, 94%; n = 4 unsure, n = 13 missing).

In terms of treatment goals, 56% (102/181) of programs indicated that they helped clients set personal consumption goals (i.e., abstinence or moderation), whereas 34% (62/181) promoted client abstinence from all use of alcohol and other drugs, and 9% (17/181) focused on abstinence specifically for substances that have caused problems (n = 9 unsure, n = 24 missing). Most programs (78%, n = 167) provided services to clients with OUD as both a primary and secondary problem; 13% (n = 28) as a primary problem only, and 5% (n = 10) as a secondary or co-occurring problem only (n = 4 unsure, n = 4 missing). One program reported that it served clients with OUD only on an urgent care basis.

Opioid addiction was identified as a major reason for treatment in a median of 25% of clients (n = 182), trailing only alcohol (50%, n = 181) and stimulants (30%, n = 171). Cannabis (20%, n = 163) and nicotine (10%, n = 120) were treated comparatively less often, while other addiction issues were treated in fewer than 10% of clients. Most opioids treated were either naturally derived and semi-synthetic opioids such as codeine, morphine, oxycodone and hydromorphone (median 15%, n = 65), or synthetic, including fentanyl, tramadol, and other opioids made in a laboratory (21%, n = 92), with a relatively small number of clients treated for heroin (6%, n = 95) or methadone (5%, n = 71).

### Proportion of Programs Offering Specific/Specialized OUD Treatment

Just over half of programs provided specific or specialized treatment for OUD clients (n = 119/205, 58%, 90% CI, 52 to 64) versus providing OUD clients the same treatment as other clients (n = 86/205, 42%, 90% CI, 36 to 48; n = 2 unsure, n = 7 missing). This varied significantly by region – 45% in prairie provinces, 50% in the Atlantic provinces, 60% in Ontario, 69% in Quebec, and 71% in BC, χ^2^(4) = 9.8, *P* < 0.05.

Among programs indicating that specialized treatment for OUD clients was provided, direct OAT provision was indicated by 77% (n = 91, 90% CI, 70 to 83), whereas 35% (n = 42, 90% CI, 28 to 43) reported some form of counselling as a specialized form of OUD treatment with no specific details on how such counselling differs from addictions counselling offered to other clients. Other specialized services for OUD clients included referral to other services providing OAT or to Rapid Access Addiction Medicine clinics (n = 18, 15%); educational, case management, or specialized harm reduction services for OUD clients (n = 16, 13%); and nonspecific or highly specialized forms of treatment such as retreats or youth services (n = 13, 11%).

### Policies and Procedures Regarding OAT

Most programs admitted clients who were participating in OAT at the time of admission (n = 173/196, 88%, 90% CI, 84 to 92; n = 11 unsure, n = 7 missing), and a minority expected or encouraged clients to discontinue OAT use prior to admission (n = 12/197, 6%, 90% CI, 4 to 10; n = 10 unsure, n = 7 missing) or taper use during treatment (n = 26/187, 14%, 90% CI, 10 to 19; n = 17 unsure, n = 10 missing). Of programs that did not admit clients currently taking OAT (n = 23, 12%, 90% CI, 8 to 16), two indicated concerns about its safety or efficacy. Ten of these 23 programs (44%, 90% CI, 26 to 62) indicated that the provision of OAT was outside the scope of their services or that they did not service a population that the informant considered appropriate for OAT provision (e.g., youth or corrections).

In terms of initiating clients on OAT, 30% of all programs surveyed (n = 59/194, 90% CI, 25 to 36) reported that they initiated clients on OAT directly. An additional 23% initiated OAT through referral to another service within their organization or agency (n = 45/194, 90% CI, 18 to 29), and 29% initiated through referral to an outside provider (n = 56/194, 90% CI, 24 to 35). The remaining 18% (n = 34/194, 90% CI, 13 to 23) did not facilitate OAT initiation at all (n = 13 unsure, n = 7 missing). Fifteen of the 18 programs not facilitating initiation (44%, 90% CI, 62 to 95) indicated that they would provide OAT, but lacked the accreditation, physician support, or other resources to effectively facilitate it.

When asked to estimate the proportion of clients with OUD receiving OAT, 150 programs provided a response, which ranged from 0% to 100%. The modal percentage was zero (n = 22, 15%) with 38% of programs estimating 10% or less and 19% indicating 90% or more. The median estimate was 28%.

Provision of OAT was compared between programs focusing on abstinence (either complete or for problematic substance use only, n = 79) versus those focusing on helping clients set personal consumption goals (n = 102; 33 missing responses). As shown in Table 1, both types of programs were equally likely to accept clients receiving OAT and to offer OAT as part of their program. However, abstinence-focused programs were more likely to require discontinuation or encourage tapering of OAT and less likely to initiate or facilitate OAT.

**Table 1.** Provision of OAT in Abstinence Versus Flexible Goal Focused Programs.

Program…	Abstinence programs	Flexible goal programs	Total	*P*
	N (%)	N (%)	N (%)	
Admits clients who are engaged in OAT	67/77 (87%)	84/95 (88%)	151/172 (88%)	0.78
Requires clients to discontinue OAT use as an admission condition	9/77 (12%)	1/98 (1%)	10/175 (6%)	0.003
Expects or encourages clients to taper use of OAT during the course of their program	16/70 (23%)	6/94 (6%)	22/164 (13%)	0.002
Program provides clients initiation on OAT?	n = 75	n = 95	N = 170	0.054^a^
Yes, program facilitates OAT	19 (25%)	30 (27%)	49 (29%)	
No, referred within organization	13 (17%)	26 (32%)	39 (23%)	
No, referred outside organization	23 (31%)	28 (30%)	51 (30%)	
Does not facilitate OAT	20 (27%)	11 (12%)	31 (18%)	

OAT: opioid agonist treatment.

^a^ Post-hoc comparison of whether or not programs facilitate OAT (either internally or externally within or outside organization) differs at *P* = 0.01.

In terms of regional differences, as shown in Table 2, there was some variability on whether programs facilitated OAT initiation, with the Prairie and Atlantic provinces slightly less likely to do so. Quebec programs were most likely to offer OAT initiation as part of their programs.

**Table 2.** Provision of OAT Regionally.

Program…	British Columbia	Prairies	Ontario	Quebec	Atlantic	Total	*P*
	N (%)	N (%)	N (%)	N (%)	N (%)	N (%)	
Admits clients who are engaged in OAT	49/54 (91%)	47/58 (81%)	39/40 (98%)	15/16 (94%)	21/26 (81%)	171/194 (88%)	0.08
Requires clients to discontinue OAT use as an admission condition	0/54	4/60 (7%)	4/39 (10%)	0/16 (0%)	3/23 (12%)	3/185 (4%)	0.11
Expects or encourages clients to taper use of OAT during their program	6/54 (11%)	9/55 (16%)	5/39 (13%)	2/15 (13%)	4/22 (18%)	23/185 (12%)	0.91
Program provides clients initiation on OAT?	n = 55	n = 58	n = 37	n = 15	n = 27	N = 192	0.001
Yes, program facilitates OAT	18 (33%)	12 (21%)	10 (27%)	8 (53%)	9 (33%)	57 (30%)	
No, referred within organization	13 (24%)	20 (35%)	3 (8%)	0 (0%)	9 (33%)	45 (23%)	
No, referred outside organization	19 (35%)	11 (19%)	17 (46%)	7 (47%)	2 (7%)	34 (18%)	
Does not facilitate OAT	5 (9%)	15 (26%)	7 (19%)	0 (0%)	7 (26%)	34 (18%)	

OAT = Opioid Agonist Treatment.

### Perceived Outcomes for OUD Clients

When asked about their perceptions of outcomes for clients with OUD versus other clients, 136 (64%) provided a response and 71 indicated that they were unsure (33%). Of those providing a response, 18% (90% CI, 12 to 24) reported that they believe that clients with OUD experience better treatment outcomes than clients with other addictions; 50% (90% CI, 43 to 57) reported a belief that they share similar outcomes, and 32% (90% CI, 26 to 40) reported that they experience worse outcomes. Overall, 42% (90% CI, 35 to 49) indicated that dropout was more likely for OUD clients than for clients presenting with other addiction problems.

As shown in Table 3, there was a significant association between perceived outcomes and whether a program actively initiated OAT and how. Better outcomes were perceived in programs that facilitated OAT through an OAT service internal to their program. Programs not conducting outcome monitoring of clients were more likely to perceive both better and worse outcomes than programs performing outcome monitoring.

**Table 3.** Variables Associated with Differential Perceived Outcomes for Clients with OUD.

Variable	Better outcomes n (%)	Similar outcomes n (%)	Worse outcomes n (%)	*P*
Program provides specialized treatment for OUD	18/25 (72%)	37/67 (55%)	23/42 (55%)	0.30
Clients with OUD are more likely to discontinue treatment than other clients	6/20 (30%)	13/55 (24%)	30/40 (75%)	0.001
Program admits clients who are engaged in OAT	22/25 (88%)	55/64 (86%)	36/41 (88%)	0.95
Program expects clients to taper off OAT	0/23 (0%)	14/61 (23%)	6/40 (16%)	0.14
Program provides clients initiation on OAT	n = 24	n = 63	n = 40	0.001
Yes, program facilitates OAT	14 (58%)	12 (19%)	10 (25%)	
No, referred within organization	5 (21%)	10 (16%)	14 (35%)	
No, referred outside organization	4 (17%)	25 (40%)	10 (25%)	
Does not facilitate OAT	1 (4%)	16 (25%)	6 (15%)	
Program's association with OAT provider	n = 22	n = 52	n = 49	0.005
Formal, within organization	20 (91%)	24 (46%)	20 (51%)	
Formal, through referral	2 (9%)	6 (12%)	5 (13%)	
Informal	0	22 (42%)	14 (36%)	
Program outcome monitoring	n = 20	n = 62	n = 39	0.07
Formal follow-up with standardized measure	6 (30%)	15 (24%)	9 (23%)	
Informal follow-up	6 (30%)	28 (45%)	8 (21%)	
Outcome monitoring not performed	8 (40%)	19 (31%)	22 (56%)	

Results shown as n (% of column). *P*-values calculated via Pearson's Chi-Square test.

OAT: opioid agonist treatment; OUD: opioid use disorder.

### Barriers and Program Needs

Fifty programs (23%, 90% CI, 18 to 28) indicated either that they need additional support to provide OAT services to clients, or that they support OAT but are experiencing other barriers to providing it to clients. This proportion did not vary regionally. Those experiencing barriers (n = 43) were asked to indicate reasons from a checklist (see Table 4). By far the most endorsed barrier was lack of onsite treatment or support staff able to prescribe OAT.

**Table 4.** Barriers Towards Provision of OAT.

Reason	n	%
Lack of on-site treatment or support staff able to prescribe OAT	32	74
Lack of safe storage capability	12	28
Inability of medical staff to access support for prescribing OAT (e.g., referrals/consultations with experts)	10	23
Insufficient access to medical resources (e.g., drugs, safe needles, overdose response kits)	10	23
Insufficient support from allied health professionals (e.g., therapists/counsellors and social workers)	9	21
Lack of knowledge or skills among medical staff to prescribe OAT	7	16
Inability of medical staff to easily access education and training opportunities	5	12
Client group that is unwilling or unprepared to initiate OAT	0	0
Other (specified)	16	37
Not accredited to provide OAT/non-medical program	7	16
Not applicable to clients (e.g., youth)	4	9
Medications not allowed on site (e.g., by laws)	2	5

OAT: opioid agonist treatment, N = 43.

### Overdose Response Kits

Eighty-six percent of programs (90% CI, 81 to 90) indicated that their program offers overdose response kits on site (n = 6 unsure, n = 13 missing). The proportion ranged from 75% in the Atlantic provinces to 96% in BC, although this did not differ statistically. Of those not providing kits on-site (n = 28), 89% (90% CI, 75 to 97) reported that they refer clients elsewhere to retrieve them, with only three programs reporting that they would not. Eighteen of 28 programs not providing kits (64%, 90% CI, 47 to 79) noted no philosophical objection to provision of overdose response kits and would do so if the resources and policy were in place to do so. The remaining 9 of 28 (32%, 90% CI, 18 to 49) did not provide kits because it was outside the scope of their program's treatment goals or simply not common practice for their program. One program reported uncertainty about why they do not provide naloxone kits.

## Discussion

More than a quarter of clients in non-residential, day, and residential psychosocial treatment programs receive treatment for OUD, with opioid use being the third most frequent presenting problem among program clients after alcohol and stimulants. Overall, the survey results demonstrate that psychosocial programs are sensitive to the need to provide programming for this population, although service enhancements are crucial. Over half of programs surveyed indicated that they provide special treatment for OUD, which was delivered primarily in the form of provision of or support for naloxone overdose kits and OAT. This varied significantly by region – 45% in prairie provinces, 50% in the Atlantic provinces, 60% in Ontario, 69% in Quebec and 71% in BC. Although those differences may reflect differences in sampling of programs in those locations, they may also flag areas where greater attention is warranted.

About one-third of programs perceived outcomes for clients with OUD to be worse than for other clients. Although we do not know why programs have this perception and whether it reflects actual outcomes, it may underpin the need for more comprehensive and specialized forms of care beyond standard clinical guidance and OAT provision. Risk of treatment dropout for clients with OUD was of particular concern with 42% of respondents reporting elevated dropout risk for OUD clients relative to others. This report suggests that treatment as currently operating requires refinement to improve client retention, and poor retention may be related to lack of staffing and a need for more critical program resources.

One of the aims of this study was to describe how OAT is being used in conjunction with psychosocial interventions to treat OUD. Encouragingly, over 80% of programs indicated a willingness to initiate clients to OAT in some fashion. This ranged from 100% of programs in Quebec to 74% in the Prairie provinces. Most programs admitted clients who were taking OAT at the time of admission and only a minority expected or encouraged clients to discontinue or taper use. However, willingness to initiate clients does not imply that most received this first line OUD treatment - the median proportion of OUD clients receiving OAT was 28% and the mode was zero. Only 30% of programs nationally had the capacity to provide OAT as part of their program structure.

A key insight gleaned was the perceived need for psychosocial addictions programs to receive further support to provide OAT in the form of training, support, knowledge, and the expertise of individuals qualified to provide and prescribe OAT to their clients. OAT was generally perceived as having observable benefit to clients. Many programs expressed a need for staff and resources to facilitate OAT, and those programs unable to offer OAT typically expressed no philosophical objections. Canadian programs may benefit from a more integrated service model in which OAT providers support under-resourced addictions programs in the form of outreach, knowledge sharing, and education that may provide necessary guidance or expertise. The need for additional staff trained and qualified to provide OAT and to have policies that support OAT on-site were also identified needs. These recommendations may also extend to supplying programs with on-site naloxone kits and providing appropriate funding for the health professionals required to offer OAT.

Perceived better treatment outcomes were described for the third of programs that facilitated OAT within their program versus making a referral to an outside service. However, this finding does not necessarily suggest that delivery of treatment with outside points of care is an ineffective model if closely coordinated. In support of this, perceived better outcomes were also associated with programs having formal (vs. informal) associations with OAT providers.

Survey respondents’ perceptions of outcomes may or may not have been based upon program outcome monitoring data. Whether or not this difference reflects biased perceptions by service providers requires objective outcome data. Whereas OAT is well studied, outcome for OUD from different psychosocial treatments or psychosocial treatment versus OAT treatment is understudied.^12^ At this point, there is insufficient evidence to guide programs in determining what psychosocial treatments are optimal for OUD.

A limitation of the study is the unknown effect of sampling bias on the overall results and representation of specific subgroups of programs within the sample. While the inclusion criteria were inclusive to maximize the overall sample size, the results must be interpreted cautiously given that the system mapping process relied on program contact information from key informants. Provincial representation was generally reflective of the population size, and thus may tend to bias toward service models employed by more populous health regions. Our group made efforts to include as many programs as possible within our pre-defined inclusion criteria. Nevertheless, our overall response rate was modest and we were unable to recruit participants in more remote parts of the country, including Yukon, Northwest Territories, and Nunavut. In addition, recruiting in Quebec was delayed by approximately 12 months due to the COVID-19 pandemic which may have influenced responses. Data collection in other provinces was completed prior to the pandemic.

Another limitation is the lack of objective outcome data to confirm program perceptions for example, in perceived outcomes and dropout rates. The data quality relied on the accuracy of the respondents who had variable amounts of objective program data upon which to make their reports. It is crucial that treatment systems move toward systematic outcome monitoring to allow identification of system strengths and weaknesses. There is little published data on the effectiveness of psychosocial treatment with individuals with OUD generally. It might be that augmentation of psychosocial treatment with OAT is unnecessary in general or with specific subgroups of clients.

A second phase of this study is exploring, through in-depth qualitative interviews with individuals who nominate their programs as “model programs” in providing service to clients with OUD. Interviews will focus on factors that these model programs identify as central to their treatment ideology and operations, with the aim of identifying best practice possibilities. In addition, CRISM is conducting a parallel study of withdrawal management services. Together, these studies will be used to synthesize knowledge translation materials and will serve as a key component of recommendations for best practices of treatment for opioid addiction.

## Supplemental Material

sj-docx-1-cpa-10.1177_07067437221082858 - Supplemental material for Treatment of Opioid Use Disorder in Canadian Psychosocial Addiction Programs: A National Survey of Policy, Attitudes, and Practice Click here for additional data file. Supplemental material, sj-docx-1-cpa-10.1177_07067437221082858 for Treatment of Opioid Use Disorder in Canadian Psychosocial Addiction Programs: A National Survey of Policy, Attitudes, and Practice by David C. Hodgins, Mathew Budd, Gail Czukar, Simon Dubreucq, Lois A. Jackson, Brian Rush, Lena C. Quilty, Denise Adams and T. Cameron Wild in The Canadian Journal of Psychiatry
