# Supplementary material for: Treatment of Opioid Use Disorder in Canadian Psychosocial Addiction Programs: A National Survey of Policy, Attitudes, and Practice
Source: Can J Psychiatry. 2022 Mar 8;67(8):638–47. doi: 10.1177/07067437221082858 (PMC9301153; doi:10.1177/07067437221082858)
Supplement: sj-docx-1-cpa-10.1177_07067437221082858 - Supplemental material for Treatment of Opioid Use Disorder in Canadian Psychosocial Addiction Programs: A National Survey of Policy, Attitudes, and Practice [file sj-docx-1-cpa-10.1177_07067437221082858.docx]

**CRISM Emerging Health Threat Survey: Treatment of Opioids in Psychosocial Programs (TOPP)**

Program & respondent info

1. Which catchment area(s) does your program serve? *(Catchment area refers to the city/county/region you draw your clients from and are legally responsible to service. If this does not apply to your program, e.g. your clients are typically referred from a national registry, please indicate the geographic area you serve.)*

- *Note that a national registry refers to any collection of client information that can assist in linking clients with specific conditions to services and/or research. This could range from simple indices, spreadsheets, or files kept by individual practitioners, to more sophisticated multi-institutional databases.*

1. Do you primarily serve an urban (population >1000) or rural population centre?
   1. Urban
   2. Rural
   3. Not sure
2. What is/are the name(s) of the program(s) you are responding on behalf of?
3. What is/are the name(s) of the organization(s) you are responding on behalf of? *(In this context, an “organization” refers to a setting for the provision of care services, which may include clinical care and/or other functions such as preventive treatment and distribution of educational materials. This might also include in-home or community services, as well as services provided by for-profit groups.)*
4. What is your position or role within the organization or program you are responding on behalf of?
5. Have you completed or will you complete this survey more than once? (E.g., for different programs.)
   1. Yes
   2. No
6. Have you forwarded our survey link to therapists/counsellors in your program? *(Only include therapists/counsellors within the program that you are responding on behalf of.)*
   1. Yes
   2. No
7. (If Q7=R1) Approximately how many therapists/counsellors did you forward the survey link to?
8. (If Q7=R2) Approximately how many therapists/counsellors do you intend on sending the survey link to?

Program details

1. Does your program provide services to clients with problematic opioid use as a primary presenting problem or a secondary/co-occurring problem? *(For the purposes of this questionnaire, opioids include the illicit drugs heroin and fentanyl, as well as prescription pain relievers such as oxycodone, hydrocodone, codeine, morphine, and any synthetic derivatives thereof. “Problematic use” is broadly defined as use of opioids that interferes with an individual’s psychosocial wellbeing or health.)*
   1. As a primary presenting problem
   2. As a secondary or co-occurring problem only (not as a primary presenting problem)
   3. Both (as either a primary or secondary problem)
   4. Neither (not admitted to this program)
   5. Not sure
2. (If Q10=R4) Why does your program not provide services to clients with problematic opioid use?
   1. Not sure

*For the following question and for several questions throughout this survey, you will be asked to provide estimates for a reporting period. This can be any 12-month period for which relevant estimates are collected, e.g., the last fiscal year, the last calendar year, or any 12-month period used by your organization for reporting purposes. There will be a question at the end of the survey to indicate which reporting period you chose to use. Note that you are* ***not*** *required to use the same reporting period for all questions, and you are free to pick any 12-month period on which to base your estimates.*

1. Approximately what percentage of clients in your program were treated for addictions to the following as a primary presenting problem during the reporting period? *(Note that this only refers to treatment received: for example, if a client is known to have multiple addictions but is not receiving treatment for them through your program, do not include this client in your estimate for the untreated addiction(s). Note that these percentages are not required to sum to 100%.)*

- *Please note that we* ***do not*** *expect exact figures for any estimates you can provide. “Best guesses” or educated approximations are encouraged.*

| Addiction | Percentage | Not sure | Not applicable |
| --- | --- | --- | --- |
| Alcohol | ______ % |  |  |
| Behavioural addictions (e.g., gambling, sex, video games) | ______ % |  |  |
| Cannabis (e.g., marijuana, hash) | ______ % |  |  |
| Hallucinogens (e.g., LSD, acid, mushrooms, PCP, Special K) | ______ % |  |  |
| Inhalants (e.g., glue, solvents) | ______ % |  |  |
| Non-alcoholic depressants (e.g., barbiturates, benzodiazepines) | ______ % |  |  |
| Opioids (e.g., heroin, codeine, morphine, fentanyl, opium) | ______ % |  |  |
| Stimulants (e.g., cocaine, crack, methamphetamine, ecstasy) | ______ % |  |  |
| Tobacco/nicotine | ______ % |  |  |
| Other *(please specify)*: | ______ % |  |  |

1. (NOT if Q10=R4) Please estimate the approximate percentage of your clients with problematic opioid use who have received treatment for addictions to the following opioids during the reporting period: *(If your organization does not track this information, please indicate ‘not sure’ for all categories.)*

| Opioid | Percentage | Not sure |
| --- | --- | --- |
| Natural opioids (Includes naturally-derived and semi-synthetic opioids (also called opiates) such as codeine, morphine, oxycodone and hydromorphone) | ______ % |  |
| Synthetic opioids (Includes fentanyl, tramadol, and other opioids made in a laboratory) | ______ % |  |
| Heroin | ______ % |  |
| Methadone | ______ % |  |
| Opium | ______ % |  |
| Other and unspecified opioids | ______ % |  |

1. (NOT if Q10=R4) Does your program provide special forms of treatment for problematic opioid use that differ from other addictions treatments you may provide? *(This may include psychosocial/counselling services, OAT, or both.)*
   1. Yes *(please specify):*
   2. No, those clients receive the same treatment as other clients
   3. Our program only serves clients with opioid use disorders
   4. Not sure
2. (NOT if Q14=R3 AND NOT if Q10=R4) In your experience, are clients being treated for opioid use disorders more likely to drop out or prematurely discontinue treatment compared to clients being treated for other forms of addiction? *(This can include clients being treated for opioid use as a primary presenting problem, or a co-occurring problem with other addictions and/or drug use.)*
   1. Yes
   2. No
   3. Not sure
3. (NOT If Q14=R3 AND NOT if Q10=R4) In your experience, do post-treatment outcomes differ between clients being treated for opioid use disorders and clients being treated for other forms of addiction?
   1. Yes, as a group these clients have better treatment outcomes *(please specify):*
   2. No, as a group these clients have poorer treatment outcomes *(please specify):*
   3. No, these clients have similar outcomes to other clients
   4. Not sure
4. Does your program provide special forms of treatment for problematic methamphetamine use that differ from other addictions treatments you may provide?
   1. Yes *(please specify):*
   2. No, those clients receive the same treatment as other clients
   3. Not applicable
   4. Not sure
5. (NOT if Q17=R3) In your experience, do post-treatment outcomes differ between clients being treated for methamphetamine use disorders and clients being treated for other forms of addiction?
   1. Yes, as a group these clients have better treatment outcomes *(please specify):*
   2. No, as a group these clients have poorer treatment outcomes *(please specify):*
   3. No, these clients have similar outcomes to other clients
   4. Not applicable
   5. Not sure

Affiliation with opioid agonist treatment (OAT) programs

*Opioid agonist treatment programs refer to those programs that treat opioid dependency through the provision of prescribed drugs such as methadone and/or Suboxone (or buprenorphine), in either an observed or take-home modality.*

1. Does your program admit clients who are receiving opioid agonist treatment (OAT) through another program or service?
   1. Yes
   2. No
   3. Not sure
2. (If Q19=R1) How many clients were admitted to your program during the reporting period who were receiving OAT at the time of admission?
   1. Not sure
3. Are clients asked to discontinue OAT before being admitted into your program?
   1. Yes *(if so, please specify why):*
   2. No
   3. Not sure
4. Do you expect clients with an opioid use disorder who are receiving OAT to taper their use of OAT during your program?
   1. Yes, this is required
   2. This is encouraged, but not required
   3. No, this is not expected and may or may not occur depending on OAT provider
   4. Not sure
5. Does your program provide clients initiation on OAT?
   1. Yes, our program provides clients initiation on OAT
   2. No, we refer clients to another program or physician within our organization that provides OAT initiation
   3. No, we refer clients to another program or physician outside of our organization that provides OAT initiation
   4. No, we do not facilitate OAT initiation
   5. Not sure
6. (If Q23=R1 or R2) How many of your clients during the reporting period were initiated on OAT through your program and/or through linkage to another program in the same organization?
   1. Not sure
7. (If Q23=R1) Has your site experienced any recent difficulties in managing OAT provision?
   1. Yes *(please specify):*
   2. No
   3. Not sure
8. Approximately how many of your clients during the reporting period has your program referred to other sites for OAT initiation?
   1. Not sure
9. What approximate total percentage of your clients with problematic opioid use are currently receiving OAT, either through your program, through outside agencies, and/or through pre-existing treatment?
   1. Not sure
10. Does your program have a formal or informal association with an OAT prescriber or program?
    1. We have a formal association with an OAT service within our program or organization *(please provide details to help explain your answer)*:
    2. We have a formal association with an OAT service provided through outside referral *(please provide details to help explain your answer):*
    3. We have an informal association with an OAT service *(please provide details to help explain your answer):*
    4. Not sure
11. (If Q23=R3 or R4) What is your organization’s philosophy on providing OAT to clients at your site? *(Select all that apply.)*
    1. We do not provide OAT because we do not believe it is an effective method of treating addiction
    2. We do not provide OAT because we have concerns about its long-term safety, adverse effects, or the risks it poses to clients
    3. We do not provide OAT but would do so if we had the means and/or resources to
    4. Providing OAT to clients is outside the scope of our program/service’s treatment goals
    5. Other *(please specify):*
    6. Not sure
12. (If Q23=R3 or R4) Are there any barriers preventing your site from offering OAT?
    1. Yes
    2. No
    3. Not sure
13. (If Q30=R1) Which of the following barriers to offering OAT apply to your site? *(Select all that apply.)*
    1. Lack of on-site treatment or support staff able to prescribe OAT
    2. Inability of medical staff to access support for prescribing OAT (e.g. referrals/consultations with experts)
    3. Insufficient support from allied health professionals (e.g. therapists/counsellors and social workers)
    4. Lack of knowledge or skills among medical staff to prescribe OAT
    5. Inability of medical staff to easily access education and training opportunities
    6. Client group that is unwilling or unprepared to initiate OAT
    7. Lack of safe storage capability
    8. Insufficient access to medical resources (e.g. drugs, safe needles, overdose response kits)
    9. Other *(please specify):*
    10. Not sure

Take-home naloxone

1. Are overdose response kits available to clients on- site? *(Note that overdose response kits can also be referred to as take-home naloxone kits.)*
   1. Yes *(please provide details to help explain your answer):*
   2. No
   3. Not sure
2. (If Q32=R1) How many overdose response kits have you dispensed during the reporting period?
   1. Not sure
3. (If Q32=R2) Do you refer clients elsewhere to retrieve overdose response kits?
   1. Yes
   2. No
   3. Not sure
4. (If Q34=R1) To where are clients referred to retrieve overdose response kits? *(Select all that apply.)*
   1. Pharmacy
   2. An outside organization providing frontline health services (e.g. walk-in clinic)
   3. Community services (e.g. harm reduction agency)
   4. Other *(please specify):*
   5. Not sure
5. (If Q32=R2) What is your organization’s philosophy on providing overdose response kits? *(Select all that apply.)*
   1. We do not provide these kits because we do not believe there is strong enough evidence of their benefit *(please provide any additional details to explain your answer, if desired):*
   2. We do not provide these kits because it is not common practice for our program *(please provide any additional details to explain your answer, if desired):*
   3. We do not provide these kits but would do so if we had the means and/or resources to
   4. Providing kits to clients is outside the scope of our program/service’s treatment goals
   5. Other *(please specify):*
   6. Not sure
6. (If Q32=R2) Are there any barriers preventing you from offering overdose response kits?
   1. Yes
   2. No
   3. Not sure
7. (If Q37=R1) Which of the following barriers to providing overdose response kits apply to your site? *(Select all that apply.)*
   1. Lack of on-site treatment or support staff able to safely dispense and provide training for kits
   2. Lack of knowledge or skills among staff to safely dispense kits and/or inform clients on their use
   3. Inability of medical staff to easily access education and training opportunities
   4. Lack of safe storage capability
   5. Insufficient access to supply
   6. Other *(please specify):*
   7. Not sure
8. Have any drug overdoses occurred on-site or among your caseload during the reporting period?
   1. Yes *(please specify the number of drug overdoses that have occurred and any other details to help explain your answer, e.g., whether this happened through a residential or non-residential service):*
   2. No
   3. Not sure

General program info

1. How is your program funded and operated?
   1. The program receives its funding primarily from a provincial or territorial health authority or government department and is operated by that same authority or department.
   2. The program receives all or the majority of its funding from a provincial or territorial health authority or government department but operates independently
   3. The program receives only partial funding from a provincial or territorial health authority or government department, but operates independently
   4. The program receives all funding from sources other than a provincial or territorial health authority or government department *(please specify):*
   5. Other *(please specify):*
   6. Not sure
2. During the reporting period, please indicate whether your program:
3. **Is promoted, tailored, or exclusively designed for certain client groups,**
4. **Accepts, but is not expressly designed for certain client groups, or**
5. **Excludes and/or provides referrals to other programs/services for certain client groups.**

| Client group | Designed for | Accepts | Excludes | Not sure |
| --- | --- | --- | --- | --- |
| Males |  |  |  |  |
| Females |  |  |  |  |
| Youth: age range ___ to ___ |  |  |  |  |
| Adults: age range ___ to ___ |  |  |  |  |
| People mandated to treatment by justice system |  |  |  |  |
| People with concurrent mental health challenges |  |  |  |  |
| Incarcerated offenders |  |  |  |  |
| First Nations, Metis, or Inuit peoples |  |  |  |  |
| LGBTQ clients |  |  |  |  |
| Other cultural groups (e.g., newcomers) |  |  |  |  |
| People with developmental disabilities (including FASD) |  |  |  |  |
| People with physical disabilities |  |  |  |  |
| Pregnant or post-partum women |  |  |  |  |
| Seniors or older adults |  |  |  |  |
| Other (please specify): |  |  |  |  |

1. Please estimate the percentage of your clients served during the reporting period who were:

|  |  | Not sure |
| --- | --- | --- |
| Persons with alcohol problems only | ______ % |  |
| Persons with other drug problems only | ______ % |  |
| Persons with both alcohol and other drug problems | ______ % |  |
| Persons with problems not related to substance abuse | ______ % |  |
| Family members/loved ones of persons with alcohol and/or drug problems | ______ % |  |
| Total | 100% |  |

1. Does your program perform biological sample testing for alcohol and/or other drug use with its clients?
   1. Yes, we conduct biological sample testing for alcohol and/or other drug use with clients in our program
   2. No, we do not perform biological sample testing for alcohol and/or other drug use with clients in our program
   3. Not sure
2. (If Q43=R1) Which of the following sample types do you use for biological sample testing? *(Select all that apply.)*
   1. Blood
   2. Breath
   3. Hair
   4. Saliva
   5. Sweat
   6. Urine
   7. Other *(please specify):*
   8. Not sure
3. (If Q43=R1) When does this testing take place? *(Select all that apply.)*
   1. Prior to intake
   2. During the course of treatment
   3. In response to acute events or specific concerns
   4. During post-treatment recovery
   5. Not sure
4. (If Q43=R1) Please indicate if this testing occurs at random or fixed intervals.
   1. Random intervals
   2. Fixed intervals *(please specify):*
   3. Not sure
5. (If Q43=R1) Are the results of biological sample testing processed immediately on-site? *(E.g., breathalyzer, rapid response laboratory testing.)*
   1. Yes
   2. No
   3. Not sure
6. (If Q43=R1) For what purpose(s) are the results of biological sample testing used? *(Select all that apply.)*
   1. Determining eligibility for admission
   2. Treatment planning
   3. Evaluating adherence to program guidelines
   4. Monitoring treatment outcomes
   5. General health screening and assessment
   6. Other *(please specify):*
   7. Not sure

Services & therapy

1. Please indicate which of the following forms of treatment or other support were offered to clients through your program during the reporting period. *(Select all that apply.)*
   1. Information about treatment or services available for mental health issues
   2. Information about treatment or services available for addictions
   3. Medication to help with mental health issues
   4. Medication to help with addictions
   5. Hospitalization overnight or longer
   6. Withdrawal management services/detoxification
   7. Residential (non-medical) treatment overnight or longer
   8. Counselling or support on a non-residential basis, including any kind of help to talk through problems related to addictions
   9. Counselling or support on a non-residential basis, including any kind of help to talk through problems related to mental health issues
   10. Responding to basic needs such as housing, finances, or food security
   11. Case management services
   12. Help to improve clients’ ability to work
   13. Education supports (e.g., to undertake self-care, to use their time, or to meet people)
   14. Help to reduce the risk of harm related to using drugs, such as needle exchanges, testing for diseases that can be passed on through drug use, etc.
   15. Crisis intervention
   16. Other *(please specify):*
   17. Not sure
2. Please indicate whether the following counselling and therapy services are offered through your program.

| Counselling or therapy service | Offered | | |
| --- | --- | --- | --- |
|  | **Yes** | **No** | **Not sure** |
| 12-step or similar support groups |  |  |  |
| Aftercare/continuing support |  |  |  |
| Contingency management |  |  |  |
| Cultural programming (e.g., sweat lodges, etc.) |  |  |  |
| Family coaching/counselling/psychotherapy |  |  |  |
| Group coaching/counselling/psychotherapy |  |  |  |
| Individual coaching/counselling |  |  |  |
| Individual psychotherapy (provided by regulated health professionals) |  |  |  |
| Relapse prevention groups |  |  |  |
| Relaxation training |  |  |  |
| Support (e.g. screening or assessment) for concurrent mental health issues |  |  |  |
| Other *(please specify):* |  |  |  |

Number of clients served per year

- *For the following set of questions, “new individuals” refer to unique clients who* ***started*** *treatment during the reporting period. This would not include individuals with an ongoing service event beginning prior to the reporting period.*
- *“Service events” refer to admissions to a particular form of service, with an associated discharge event or case closure completed within the reporting period.*
  - *Note that this is not concretely equivalent to number of appointments. A client may have many appointments for treatment which would fall under the umbrella of a single service event (e.g., admission and discharge from a day program).*
- *For the purposes of this survey, a client who transfers between levels of service, e.g., enrolling in a day program after receiving non-residential care through outpatient services, would be associated with two different service events.*
- *Provision of support to loved ones of persons with substance use disorders should be counted as part of service event estimates, if applicable.*
- *Please note that we do* ***not*** *expect exact figures for any estimates you can provide. “Best guesses” or educated approximations are encouraged.*

1. Did your program provide non-residential services for clients during the reporting period for alcohol and/or other drug use? *(This could be provided on a recurring schedule, e.g., 1-2 hours per week.)*
   1. Yes
   2. No
   3. Not sure
2. (If Q51=R1) How many new individuals received non-residential care for alcohol and/or other drug use during the reporting period?
   1. Not sure
3. (If Q51=R1) How many service events were provided for alcohol and/or other drug use on a non-residential basis during the reporting period?
   1. Not sure
4. (If Q51=R1) What is the approximate capacity of your non-residential service on a monthly basis?
   1. Not sure
5. Did your program provide day or evening treatment for clients during the reporting period for alcohol and/or other drug use? *(Day treatment is any intensive, structured non-residential treatment, typically provided five days/evenings per week for several hours per day, e.g., 3-4 hours every weekday.)*
   1. Yes
   2. No
   3. Not sure
6. (If Q55=R1) How many new individuals received day or evening treatment for alcohol and/or other drug use during the reporting period?
   1. Not sure
7. (If Q55=R1) What is the approximate capacity of your day or evening treatment service on a monthly basis?
   1. Not sure
8. Did your program provide residential service for clients during the reporting period for alcohol and/or other drug use? *(Residential service is any treatment or rehabilitation service provided to clients while they reside in-house; this can be short term, e.g., 40 days or less, or long term, e.g., longer than 40 days.)*
   1. Yes
   2. No
   3. Not sure
9. (If Q58=R1) Please indicate the number of beds designated for treatment of clients with alcohol and/or drug addictions.
   1. Not sure
10. (If Q58=R1) Please estimate the average number of beds in use by clients at any given time (i.e., average occupancy rate) that are designated for treatment of alcohol and/or drug problems.
    1. Not sure
11. (If Q58=R1) How many new individuals received residential services for alcohol and/or other drug use during the reporting period?
    1. Not sure
12. (If Q58=R1) How many service events (i.e., number of admissions with associated discharges) were provided during the reporting period for alcohol and/or drug use on a residential basis?
    1. Not sure
13. (If Q58=R1) What was the average length of stay for alcohol and/or other drug problems for clients admitted to residential services during the reporting period?
    1. Not sure

Program treatment goals & philosophy

1. Which one statement below best reflects the program’s long-term client goals regarding substance use?  *(Note that this question is not referring to substance use during treatment, which may differ from long-term goals, e.g., residential program rules.)*
   1. The program focuses on client abstinence from all use of alcohol and other drugs
   2. The program focuses on client abstinence only from use of alcohol and other drugs that have caused problems
   3. The program focuses on helping clients set personal consumption goals (abstinence or moderate use)
   4. Not sure
2. What is your program’s policy on clients abstaining from use of nicotine or tobacco as part of their long-term goals?
   1. Abstinence from nicotine or tobacco is not required
   2. Abstinence from nicotine or tobacco is encouraged but not required
   3. Abstinence from nicotine or tobacco is required
   4. Not sure
3. (If Q64=R1) Please indicate which of the following psychoactive substances clients are advised to abstain from as part of their long-term goals, in addition to alcohol and other drugs. *(Select all that apply.)*
   1. Caffeine
   2. Cannabis
   3. Prescribed psychoactive medications for mental health (e.g., anxiety medication)
   4. Prescribed opioids (e.g., as for chronic pain)
   5. Other *(please specify):*
   6. Not sure
4. Which of the following statements most closely matches the definition of recovery used in this program in terms of substance use? (*Please select one response only.*)
   1. No use of any substance – drugs or alcohol
   2. No use of any substance – drug or alcohol – except as prescribed by your doctor
   3. No use of substance of choice but some use of other substances
   4. Moderate or controlled use of any substance – drug or alcohol
   5. Moderate or controlled use of alcohol
   6. Moderate or controlled use of drugs
   7. Not sure
5. Which of the following statements are reflected in your program’s concept of holistic recovery? *(Select all that apply.)*
   1. Achieving abstinence from alcohol or other drugs
   2. Improved quality of life *(for the purposes of this survey, quality of life refers to a subjective state of physical and emotional well-being)*
   3. Absence of thoughts or cravings for alcohol or other drugs
   4. Client maintains their pre-defined treatment goals
   5. Our program does not have a set definition of recovery
   6. None of the above *(please specify):*
   7. Not sure

1. Does your program provide clients with harm reduction services focused on reducing client substance use? *(Harm reduction refers to an evidence-based approach to treatment that minimizes risks and harms associated with substance use that do not explicitly call for abstention from substance use, e.g., overdose prevention plans and peer leadership groups.)*
   1. Yes *(please provide details, if desired):*
   2. No *(please provide details, if desired):*
   3. Not sure
2. Does your program employ trauma-informed practices? *(Trauma-informed practices refer to an organizational philosophy guided by a set of principles that recognize and prioritize the client’s safety, comfort, and empowerment. This philosophy is informed by an understanding of the links between substance use and traumatic experiences. Note that the scope of trauma-informed practice should be inclusive of every aspect of service delivery, and not simply guide the direct provision of treatment: administration, management, resource allocation, program planning and evaluation, etc. should all be trauma-informed to fit this description.)*
   1. Yes
   2. No
   3. Not sure
3. Does your program employ a means of providing cultural adaptations to care and/or other ways of acknowledging and attending to patient diversity?
   1. Yes *(please specify):*
   2. No
   3. Not sure

Admissions/discharge policies

1. Which of the following methods are administered to determine whether potential clients are admitted to your program? *(Select all that apply).*
   1. Structured interviews *(e.g., standardized protocols or formats for assessment)*
   2. Unstructured interviews
   3. Standardized psychological testing
   4. Biological sample testing
   5. Structured tests for alcohol and/or other drug use *(e.g., questionnaires)*
   6. Other *(please specify):*
   7. None
   8. Not sure
2. Which of the following conditions would result in a client being refused entry into your program? *(Select all that apply.)*

**Client…**

- 1. Is intoxicated at the time of requesting service
  2. Has not been substance-free for a sufficient period of time
  3. Has goals besides abstinence
  4. Is not self-referred
  5. Does not have a physician referral
  6. Is taking prescription medication
  7. Is currently using other drugs
  8. Is taking restricted medications (e.g., opioids)
  9. Is medically unstable
  10. Is not sufficiently motivated to make changes
  11. Experiences problematic use of multiple substances
  12. Has a concurrent mental health issue
  13. Has a concurrent medical issue
  14. Exhibits acute suicidality
  15. Is currently involved in the legal system
  16. Does not have stable housing
  17. Does not have a current family doctor or GP
  18. Other *(please specify):*
  19. Not sure

1. (If Q73=R6) Which prescription medication(s) would result in a client being refused entry to the program?
   1. Not sure
2. What forms of support or referral to other care facilities/programs are offered to clients who do not meet the criteria for admission to your program? *(Select all that apply.)*
   1. Information is offered to the client about other program/services
   2. Connect or refer clients directly with other program/services
   3. None
   4. Other *(please specify):*
   5. Not sure
   6. Not applicable
3. Which of the following services are provided to clients if the program or facility has reached maximum capacity? *(Select all that apply.)*

**Clients are…**

- 1. Placed on a waiting list
  2. Referred directly to other programs
  3. Referred directly to other facilities that offer the same program
  4. Provided information about other programs or facilities
  5. Other (please specify):
  6. Our program does not have a policy for such situations
  7. Not sure

1. Which of the following conditions would result in a client being discharged prior to completing treatment? *(Select all that apply.)*

**Client…**

- 1. Shows lack of engagement in program
  2. Has poor attendance
  3. Shows no evidence of progress or improvement
  4. Is disruptive or interfering with other clients
  5. Is violent or threatening
  6. Uses non-approved substances during treatment
  7. Has medical needs that are interfering with program effectiveness and cannot be met within this program
  8. Has addiction needs that are interfering with program effectiveness and cannot be met within this program
  9. Has mental health needs that are interfering with program effectiveness and cannot be met within this program
  10. Is in an emotional state that is interfering with program effectiveness and cannot be met within this program
  11. Other *(please specify):*
  12. Not sure

1. (If Q77=R4) What kinds of behaviours would be considered disruptive and potentially result in a client being discharged? *(Select all that apply.)*
   1. Swearing
   2. Excessive argumentativeness
   3. Resistance or refusal to comply with rules and requests
   4. Deliberately aggravating or showing aggression toward clients or service staff
   5. Talking over others
   6. Other *(please specify):*
   7. Not sure
2. What forms of support or referral to other care facilities/programs are offered to clients who are discharged prior to completing treatment? *(Select all that apply.)*
   1. Information is offered to the client about other program/services
   2. Connect or refer clients directly with other program/services
   3. None
   4. Other *(please specify):*
   5. Not sure

Outcomes & follow-up

1. Please indicate which of the following options most closely matches your program’s policy on client follow-up monitoring of treatment outcomes:
   1. We conduct formal follow-up outcome monitoring (e.g., through use of a standardized instrument to quantitatively assess outcomes and develop evidence-based service delivery models)
   2. We informally follow-up with clients to assess outcome (e.g., collecting over-the-phone feedback on treatment experiences)
   3. Follow-up of clients is not typically performed
   4. Not sure
2. (If Q80=R1 or R2) For which clients do you perform follow-up?
   1. For all clients
   2. Only for clients completing the program
   3. For a subsample of clients
   4. Not sure
3. (If Q80=R1 or R2) When do these assessments take place? *(Select all that apply.)*
   1. Prior to intake to establish a baseline
   2. During the course of treatment
   3. At time of discharge or exit from the program, irrespective of treatment completion
   4. At successful treatment completion
   5. At regular intervals post-treatment *(please specify):*
   6. During the period that clients attend a clinical aftercare program
   7. Not sure
4. (If Q82=R2) Please indicate if this testing occurs at random or fixed intervals.
   1. Random intervals
   2. Fixed intervals *(please specify):*
   3. Not sure
5. (If Q80=R1 or R2) How are assessments of treatment outcomes conducted? *(Select all that apply.)*
   1. Telephone interview with clinical staff
   2. Telephone interview with support staff
   3. Telephone interview with independent third party *(please explain):*
   4. Telephone interview with volunteer
   5. Online link provided to client
   6. Other *(please specify):*
   7. Not sure
6. (If Q80=R1 or R2) Are treatment outcomes summarized in the form of a written report?
   1. Yes
   2. No
   3. Not sure
7. (If Q85=R1) Which of the following pieces of information are collected in these reports? *(Select all that apply.)*
   1. Standard demographic information (e.g., age and sex)
   2. Scores on standardized instruments (if applicable)
   3. Program participation metrics (e.g., attendance and dropouts)
   4. Outcomes at the point of discharge (e.g., end of treatment substance use)
   5. Post-program and aftercare outcomes
   6. Other *(please specify):*
   7. Not sure

Reporting period

1. Was the same reporting period used for all the statistical and estimation questions in this survey?
   1. Yes (please indicate the reporting period used):
   2. No (please explain):

Optional comments & feedback

*As part of this project, we are looking for examples of programs with effective models for serving individuals with opioid use disorder. The following set of questions would assist us in identifying such programs.*

1. Do you consider your program to be a model program for treating or otherwise supporting individuals with opioid dependence?
   1. Yes, we are a model program
   2. We do our best but are not a model program
   3. We need to do better
2. Please elaborate on your answer above, if possible.
3. Can you identify another program that you believe is a model program? *(If so, please explain.)*
4. Do you have any additional thoughts about the treatment of individuals with opioid use disorder in psychosocial treatment programs that you would like to share?
5. Did you experience any technical issues or interruptions while taking this survey? *(If so, please explain.)*
6. If you have any other suggestions, questions, or comments that would assist us in improving this survey, please outline them here
